# Supplementary material for: Reevaluating the true diagnostic accuracy of dipstick tests to diagnose urinary tract infection using Bayesian latent class analysis
Source: PLoS One. 2020 Dec 31;15(12):e0244870. doi: 10.1371/journal.pone.0244870 (PMC7774958; doi:10.1371/journal.pone.0244870)
Supplement: S1 File — (DOCX) [file pone.0244870.s001.docx]

**A simplified explanation of Bayesian latent class modelling: Three tests in one population model**

In the context of diagnostic test evaluation, the study sample can be considered as a mixture of 2 latent classes—one with the target condition and the other without the target condition. Relative proportion of the latent class with the target condition would correspond to the prevalence, which is an unobserved parameter. If the total number of patients in the study sample is ‘n’, the number of patients in the latent class with the target condition (D) can be expressed as n*prevalence, and the number of patients in the latent class without the target condition (ND) can be expressed as n*(1−prevalence). Within a latent class, the observed frequency of a given combination of 3 test results can be expressed by multiplying the probabilities of observing individual test results, using the multiplication rule of probability, provided that the tests in question are independent. Say, if 1 indicates a positive test result and 0 indicates a negative test result, the probability of observing a combination of all three tests positive (test profile 111) can be obtained by multiplying the individual probabilities of positive result for the 3 tests. (See below for more on independence assumption)


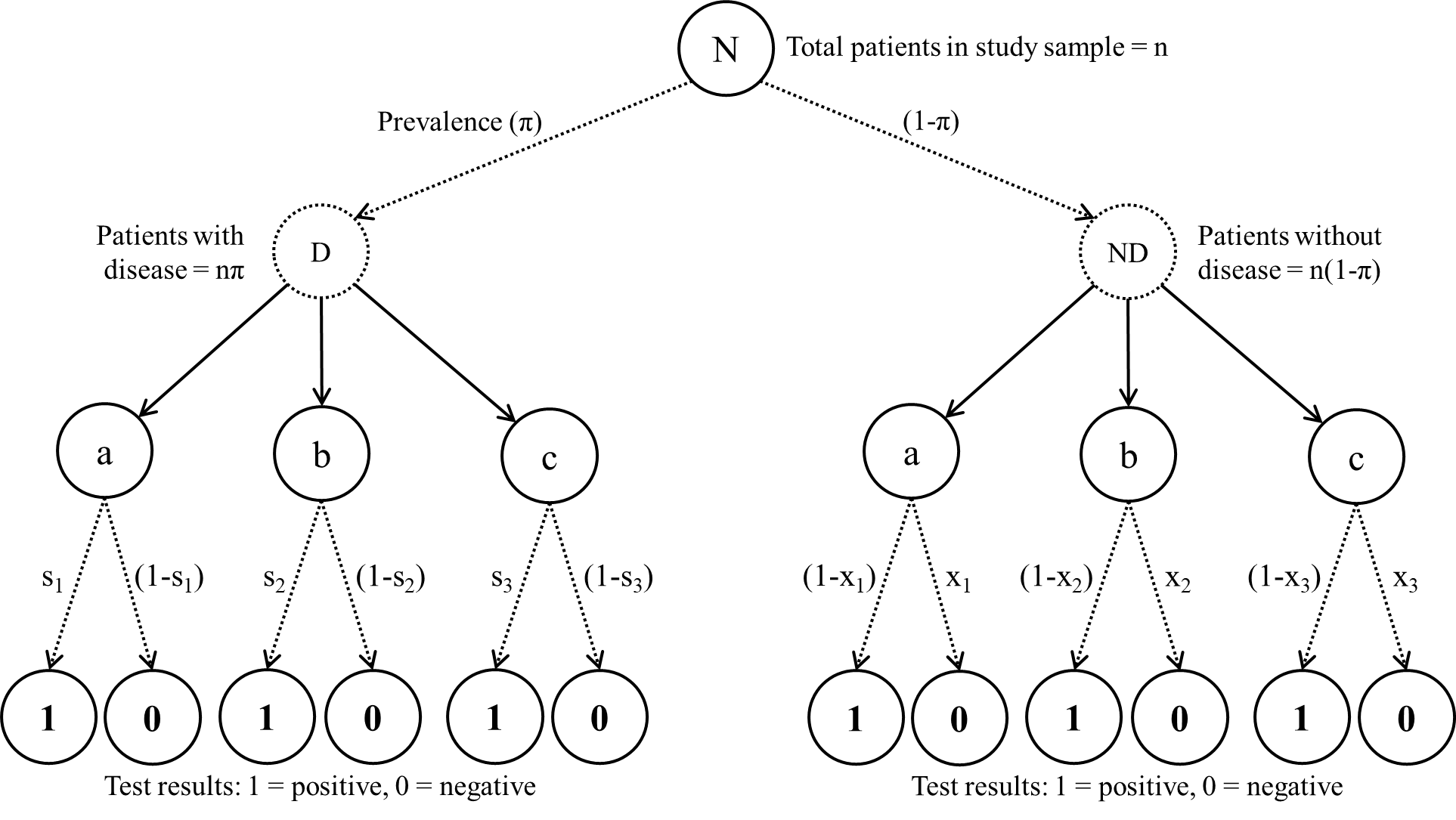


**S1 Figure. Schematic depiction of all possible outcomes when 3 independent diagnostic tests are applied to 1 study sample.** Unknown parameters are depicted by interrupted lines. a,b,c represent individual tests; s_1,2,3_=sensitivity; x_1,2,3_=specificity. (Adapted from: Reference 24)

However, the study sample contains 2 latent classes. Therefore, observed frequency of positive results in the study sample for any test would be a total of (true positives + false positives), and observed frequency of negative results for any test would be a total of (true negatives + false negatives). Further,

- True positives can be expressed as, sensitivity*n*prevalence
- True negatives as, specificity*n*(1-prevalence)
- False negatives as, (1-sensitivity)*n*prevalence
- False positives as, (1-specificity)*n*(1-prevalence)

Thus, the total number of observations with the test profile ‘111’ (ie, all 3 tests positive) can be modelled as,


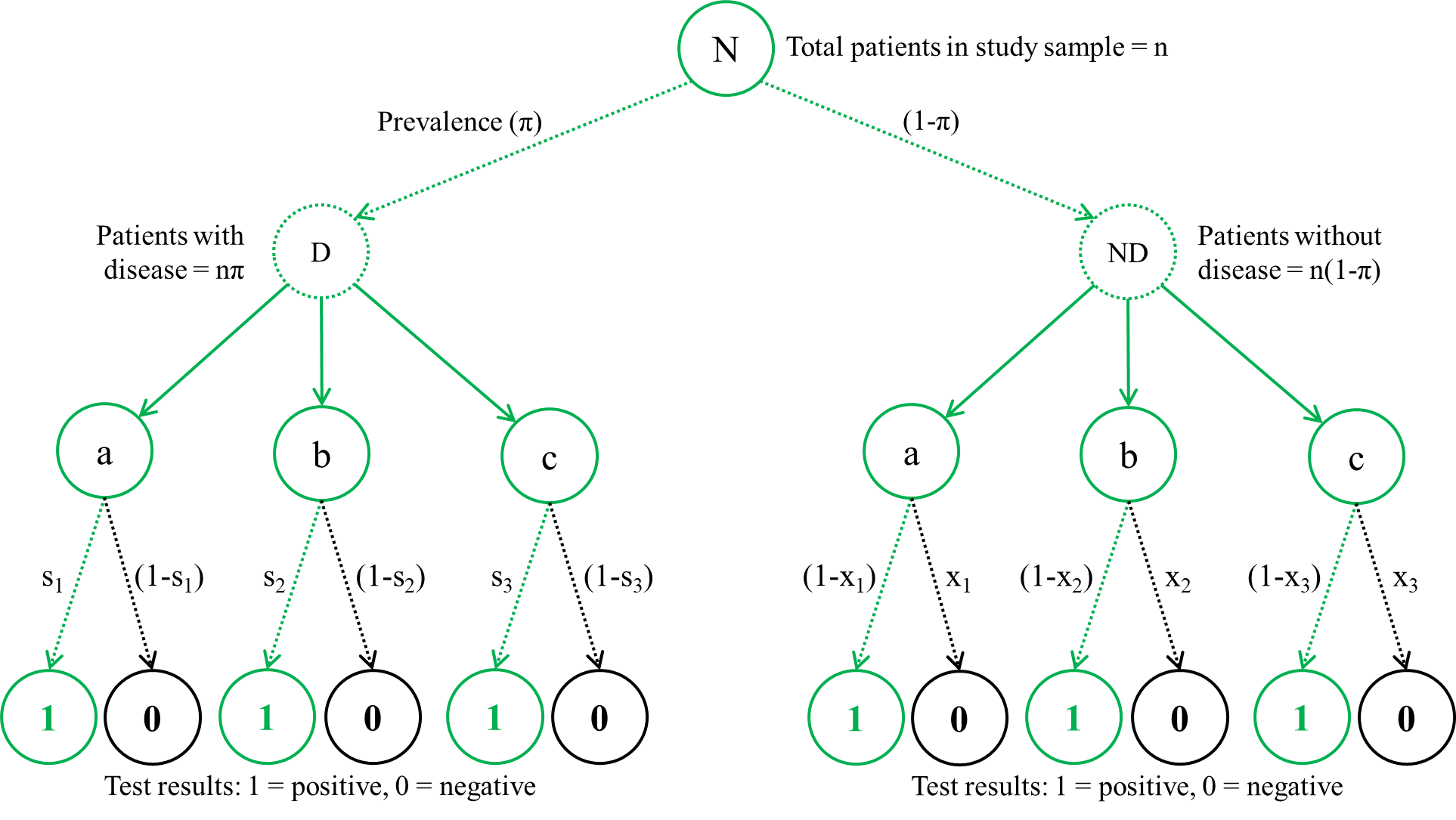


**S2 Figure. Modelling test profile 111.** Relevant probabilities are highlighted green.

Total positives = True positives + False positives

= (nπ*(s_1_*s_2_*s_3_)) + (n(1-π)*((1-x_1_)*(1-x_2_)*(1-x_3_))) ……….See Figure 2.

where, n=total number of observations in the study sample; π=prevalence; s_1,2,3_=sensitivity; x_1,2,3_=specificity; a,b,c represent individual tests. nπ would correspond to patients with target condition and n(1-π) would correspond to those without the target condition. Therefore, (nπ*(s_1_*s_2_*s_3_)) would depict true positives among those with the test profile 111, and (n(1-π)*((1-x_1_)*(1-x_2_)*(1-x_3_))) would depict false positives among those with the test profile 111.

Likewise, the total number of observations with the test profile ‘000’ (ie, all 3 tests negative) can be modelled as,


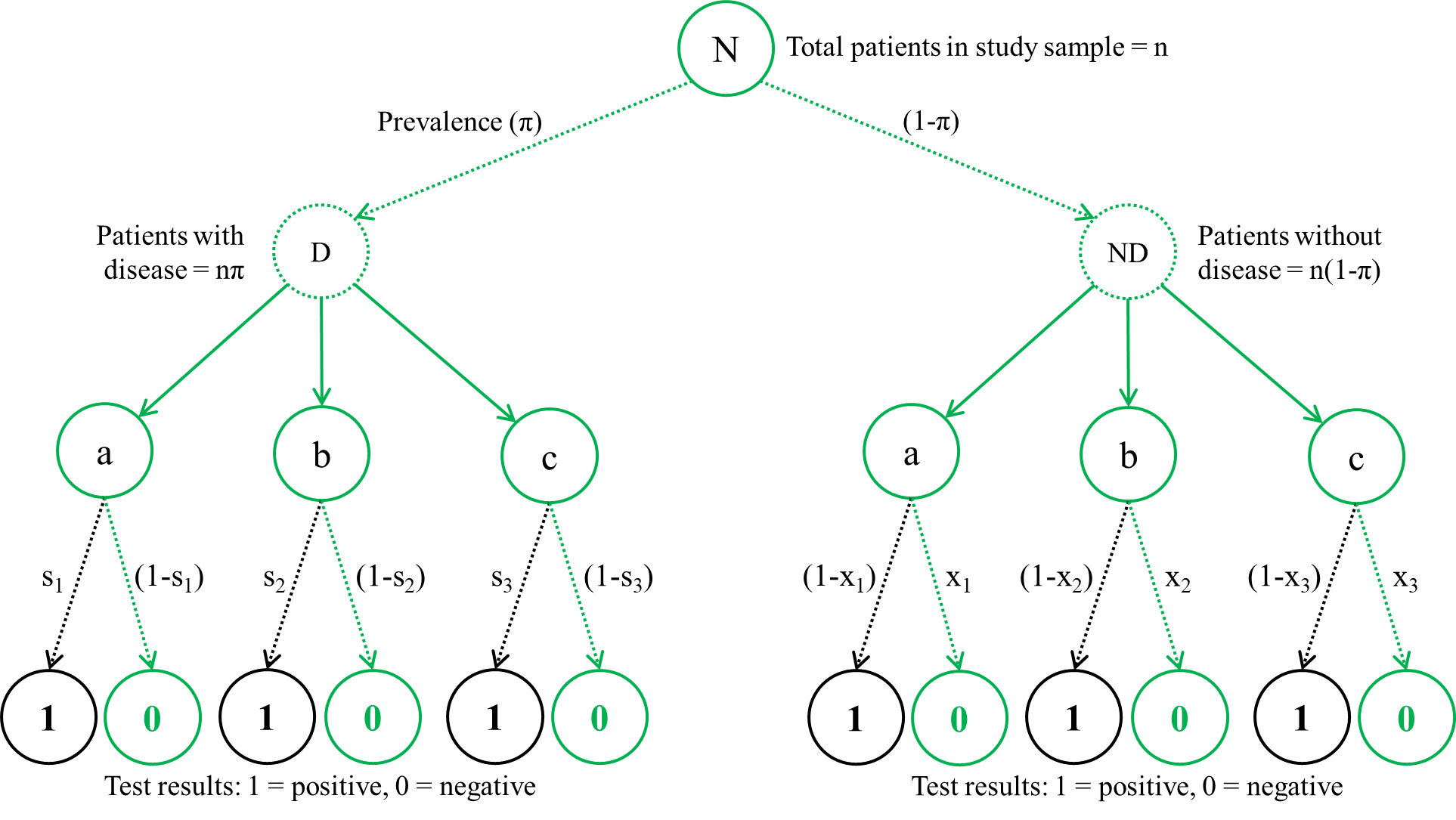


**S3 Figure. Modelling test profile 000.** Relevant probabilities are highlighted green.

Total negatives = False negatives + True negatives

= (nπ*((1-s_1_)*(1-s_2_)*(1-s_3_))) + (n(1-π)*(x_1_*x_2_*x_3_)) ………….See Figure 3.

where, n=total number of observations in the study sample; π=prevalence; s_1,2,3_=sensitivity; x_1,2,3_=specificity; a,b,c represent individual tests. nπ would correspond to patients with target condition and n(1-π) would correspond to those without the target condition. Therefore, (nπ*((1-s_1_)*(1-s_2_)*(1-s_3_))) would depict false negatives among those with the test profile 000, and (n(1-π)*(x_1_*x_2_*x_3_)) would depict true negatives among those with the test profile 000.

In the context of 3 different tests performed simultaneously, 2^3^=8 distinct test profiles are possible (a multinomial experiment), as given in the table below.

| **Observed response vector of individual tests** | | | **Test profile** |
| --- | --- | --- | --- |
| **a[]** | **b[]** | **c[]** |  |
| 1 | 1 | 1 | 111 |
| 1 | 1 | 0 | 110 |
| 1 | 0 | 1 | 101 |
| 0 | 1 | 1 | 011 |
| 1 | 0 | 0 | 100 |
| 0 | 1 | 0 | 010 |
| 0 | 0 | 1 | 001 |
| 0 | 0 | 0 | 000 |

**S1 Table. All possible combination of test results (test profiles) when 3 independent tests are simultaneously done on 1 study sample.**

The above two profile-specific equations can be combined into a generic equation, as given below, so that the program repeats the model (called ‘repeated structures’) for all 8 test profiles (called ‘loop variable’). Incorporating the test result (called ‘observed response vector’) as 0 or 1 into the equation would retain only appropriate elements for a given test profile and eliminate inappropriate elements in the model.

Probability of *i*^th^ test profile comprised of a combination of 3 independent test results can be modelled as,

p[*i*]<-(nπ*positive[*i*]) + (n(1-π)*negative[*i*])

Total positives among those with the test profile *i* can be modelled as:

positive[*i*] = (s_1_*a[*i*]+(1-s_1_)*(1-a[*i*])) * (s_2_*b[*i*]+(1-s_2_)*(1-b[*i*])) * (s_3_*c[*i*]+(1-s_3_)*(1-c[*i*]))

Total negatives among those with the test profile i can be modelled as:

negative[*i*] = ((1-x_1_)*a[*i*]+x_1_*(1-a[*i*])) * ((1-x_2_)*b[*i*]+x_2_*(1-b[*i*])) * ((1-x_3_)*c[*i*]+x_3_*(1-c[*i*]))

n=total number of observations in the study sample; π=prevalence; s_1,2,3_=sensitivity; x_1,2,3_=specificity; a,b,c represent individual tests. a[i],b[i],c[i] represent the test result (positive or negative, coded as 1 or 0) on a given profile *i*, as given in the table above.

Thus, the probability of the observed test profile is expressed as a function of the sensitivity, specificity, and prevalence parameters. Then, the unknown parameters π, s_1_, s_2_, s_3_, x_1_, x_2_, and x_3_ are estimated from the models and the observed data using a Bayesian approach. In the Bayesian approach to latent class analysis, unknown parameters are considered as random variables, each following a probability distribution. Prior knowledge about the unknown parameters, if available, is specified as a prior probability distribution, which is then combined with information from the observed data to obtain a posterior probability distribution for each parameter, which can be used to obtain point estimates of these unknown parameters with 95% credible intervals (Bayesian confidence intervals). In the absence of prior knowledge, one could still conduct a Bayesian analysis by assuming that nothing is known about the parameters in question (known as non-informative priors). Although many prior studies are available on the diagnostic accuracy of urine dipstick tests, these studies have found wide variation in the estimates of sensitivity and specificity. Therefore, we chose to assume non-informative priors.

In the present study, beta distribution with parameters (0.5, 0.5) was used to indicate non-informative priors for all unknown parameters, which implies that every value of the unknown parameter is equally likely prior to the analysis, i.e. we knew nothing about the diagnostic tests before the data set was analysed. However, a constraint was introduced that the sensitivity and specificity were >0.40 (known as truncation). Otherwise, the model might predict the accuracy of the test the other way around (predicting the sensitivity as 10% and specificity as 10% of a test with a true sensitivity of 90% and true specificity of 90%).

Markov chain Monte Carlo (MCMC) is a Bayesian method used to estimate the unknown parameters and their 95% credible intervals, by a process described as sampling from the posterior. Each Markov chain is run 20,000 iterations with 5,000 burn-in iterations. Initial iterations before the MCMC chains converge do not yield accurate estimates, and hence are discarded. These discarded iterations are called burn-in iterations. Running more than 1 Markov chain helps check the convergence of MCMC simulations. If both the chains overlap each other, one can be reasonably sure about convergence. Once convergence has been achieved, the simulation is run for a further number of iterations to obtain samples for posterior inference. The posterior samples may be summarised either graphically or numerically, by calculating summary statistics.

**A note on conditional independence assumption**

One key assumption in the above approach is that the imperfect tests under study tend to misclassify patients independently. It means that a positive or negative test result with any of the tests is entirely dependent only on the true disease status, and it is not influenced by the results of other tests (known as conditional independence) (Adapted from: Reference 21). Sensitivity and specificity might be overestimated if the tests tend to misclassify the same patients (known as positive dependence). Empirical verification of this assumption is very challenging. Tests based on different biological principles are unlikely to misclassify the same patients and hence are expected to be conditionally independent (Kostoulas P, Nielsen SS, Branscum AJ, Johnson WO, Dendukuri N, Dhand NK, et al. STARD-BLCM: Standards for the Reporting of Diagnostic accuracy studies that use Bayesian Latent Class Models. Prev Vet Med. 2017;138:37-47). In the present case, while urine culture directly captures the presence of pathogenic bacteria in the urine, leukocyte esterase positivity captures the host response to infection, and urinary nitrite testing assesses the biochemical interaction between some of the pathogenic bacteria and the host milieu. Therefore, we expected that misclassification by these 3 tests would be conditionally independent.

**Two tests in two populations model**

The same principles discussed until now can be extended to this situation.


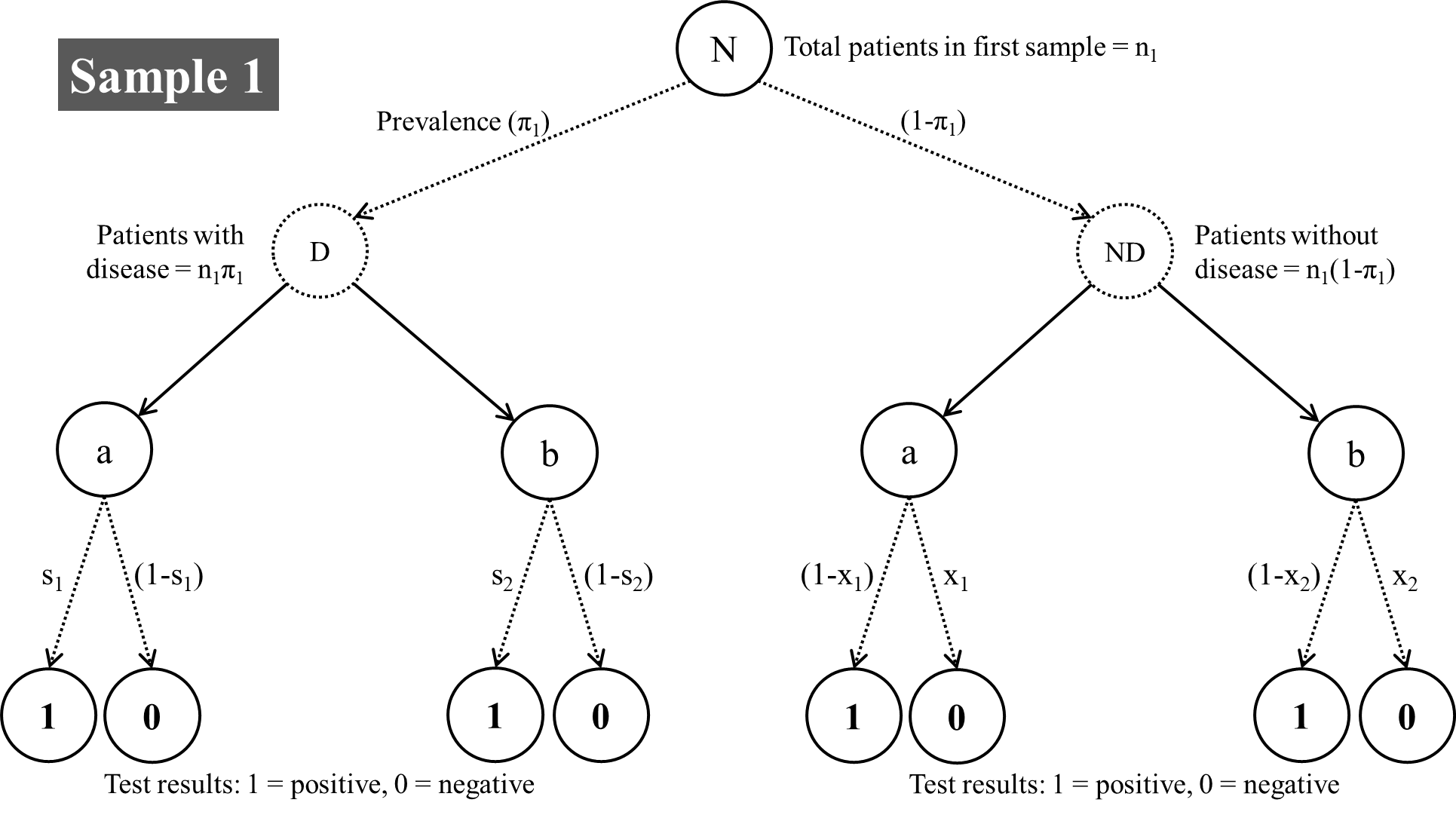


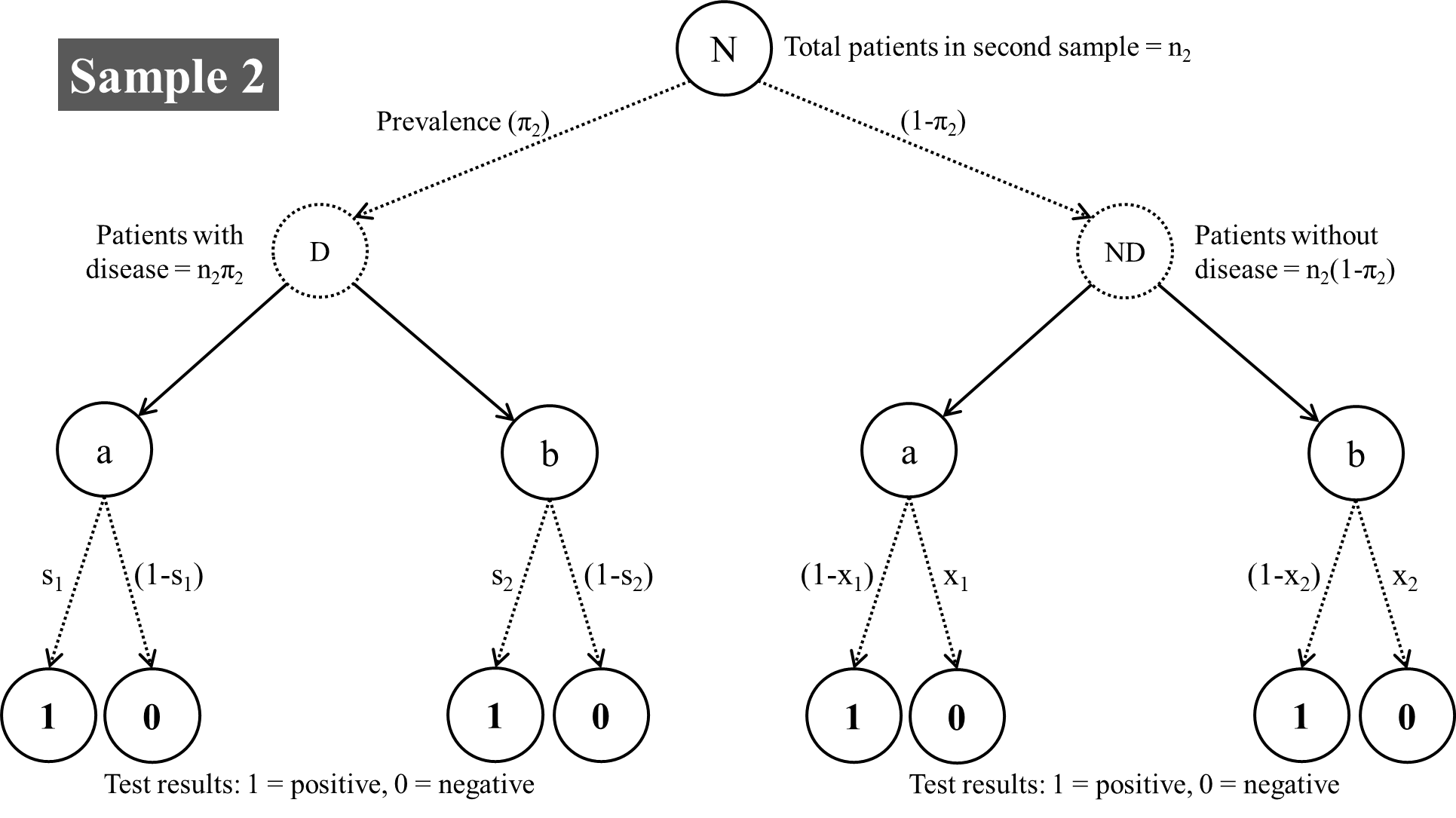


**S4 Figure. Schematic depiction of all possible outcomes when 2 independent diagnostic tests are applied to 2 study samples with varying prevalence (π_1_ and π_2_).** Unknown parameters are depicted by interrupted lines. a,b represent individual tests; s_1,2_=sensitivity; x_1,2_=specificity.

In the context of 2 different tests performed simultaneously, 2^2^=4 distinct test profiles are possible in each of the study samples, as given in the table below.

| **Observed response vector of individual tests** | | **Test profile** |
| --- | --- | --- |
| **a[]** | **b[]** |  |
| **Study sample 1** | | |
| 1 | 1 | 11 |
| 1 | 0 | 10 |
| 0 | 1 | 01 |
| 0 | 0 | 00 |
| **Study sample 2** | | |
| 1 | 1 | 11 |
| 1 | 0 | 10 |
| 0 | 1 | 01 |
| 0 | 0 | 00 |

**S2 Table. All possible combination of test results (test profiles) when 2 independent tests are simultaneously done on 2 study samples.**

Probabilities of *i*^th^ test profile comprised of a combination of 2 independent test results in the 2 study samples can be individually modelled as,

p1[*i*]<-(n_1_π_1_*positive[*i*]) + (n_1_(1-π_1_)*negative[*i*])

p2[*i*]<-(n_2_π_2_*positive[*i*]) + (n_2_(1-π_2_)*negative[*i*])

Total positives among those with the test profile *i* can be modelled as:

positive[*i*] = (s_1_*a[*i*]+(1-s_1_)*(1-a[*i*])) * (s_2_*b[*i*]+(1-s_2_)*(1-b[*i*]))

Total negatives among those with the test profile i can be modelled as:

negative[*i*] = ((1-x_1_)*a[*i*]+x_1_*(1-a[*i*])) * ((1-x_2_)*b[*i*]+x_2_*(1-b[*i*]))

**Model specification in WinBUGS program**

**3 tests in 1 population model**

# Imperfect gold standard - 3 tests applied to a single population

# Multinomial and conditional independence model

model{

total<-sum(freqobs[1:8])

# Likelihood

freqobs[1:8]~dmulti(p[1:8],total)

for (i in 1:8){

p[i]<-prev*(positive[i])+(1-prev)*(negative[i])

positive[i]<-(s[1]*a[i]+(1-s[1])*(1-a[i])) * (s[2]*b[i]+(1-s[2])*(1-b[i]))

* (s[3]*c[i]+(1-s[3])*(1-c[i]))

negative[i]<-((1-x[1])*a[i]+x[1]*(1-a[i])) * ((1-x[2])*b[i]+x[2]*(1-b[i]))

* ((1-x[3])*c[i]+x[3]*(1-c[i]))

}

# Prior

prev~dbeta(0.5,0.5)

for (j in 1:3){

s[j]~dbeta(0.5,0.5)I(0.4,)

x[j]~dbeta(0.5,0.5)I(0.4,)

}

# Prediction

freqpred[1:8]~dmulti(p[1:8],total)

#Bayesian p value

for (i in 1:8){

pvalue[i]<-step(freqpred[i]-freqobs[i])

}

# PPV-NPV

for (j in 1:3){

ppv[j]<- s[j]*prev / (s[j]*prev + (1-x[j])*(1-prev))

npv[j]<- x[j]*(1-prev) / (x[j]*(1-prev) + (1-s[j])*(prev))

}

**2 tests in 2 populations model**

# Imperfect gold standard - 2 tests applied to 2 populations

model{

total[1]<-sum(freqobs[1:4])

total[2]<-sum(freqobs[5:8])

# Likelihood

freqobs[1:4]~dmulti(p1[1:4],total[1])

freqobs[5:8]~dmulti(p2[1:4],total[2])

for (i in 1:4){

p1[i]<-prev[1]*(positive[i])+(1-prev[1])*(negative[i])

}

for (i in 1:4){

p2[i]<-prev[2]*(positive[i])+(1-prev[2])*(negative[i])

}

for (i in 1:4){

positive[i]<-(s[1]*a[i]+(1-s[1])*(1-a[i])) * (s[2]*b[i]+(1-s[2])*(1-b[i]))

negative[i]<-((1-x[1])*a[i]+x[1]*(1-a[i])) * ((1-x[2])*b[i]+x[2]*(1-b[i]))

}

# Prior

for (j in 1:2){

prev[j]~dbeta(0.5,0.5)

s[j]~dbeta(0.5,0.5)

x[j]~dbeta(0.5,0.5)I(0.4,)

}

# Prediction

freqpred[1:4]~dmulti(p1[1:4],total[1])

freqpred[5:8]~dmulti(p2[1:4],total[2])

#Bayesian p value

for (i in 1:8){

pvalue[i]<-step(freqpred[i]-freqobs[i])

}

# PPV-NPV

for (j in 1:2){

ppv[j]<- 100*(s[j]*prev[1]*total[1]+s[j]*prev[2]*total[2]) / ((s[j]*prev[1]*total[1]+s[j]*prev[2]*total[2]) + ((1-x[j])*(1-prev[1]*total[1])+(1-x[j])*(1-prev[2])*total[2]))

npv[j]<- 100*(x[j]*(1-prev[1])*total[1]+x[j]*(1-prev[2])*total[2]) / ((x[j]*(1-prev[1])*total[1]+x[j]*(1-prev[2])*total[2]) + ((1-s[j])*(prev[1])*total[1]+(1-s[j])*(prev[2])*total[2]))

se[j]<-s[j]*100

sp[j]<-x[j]*100

prevalence[j]<-prev[j]*100

}

}
