# Supplementary material for: Reevaluating the true diagnostic accuracy of dipstick tests to diagnose urinary tract infection using Bayesian latent class analysis
Source: PLoS One. 2020 Dec 31;15(12):e0244870. doi: 10.1371/journal.pone.0244870 (PMC7774958; doi:10.1371/journal.pone.0244870)
Supplement: S2 File — (PDF) [file pone.0244870.s002.pdf]

Result Page

Model Code : MODEL103

Model Name : The 3-tests in 1-population Model (Simplified Interface)

Job ID : 20191117175015047

1. Summary Table

Prevalence, sensitivities and specificities, positive and negative predictive values (PPV and NPV) estimated by using gold standard model and Bayesian latent class model (LCM). **(Hide)**

Parameters	Test A was assumed as a perfect gold standard (%)*	Bayesian latent class model (%) **
**Prevalence**	40.3 (32.4 - 48.6)	60.6 (47.4 - 83.4)
**Test A**		
Sensitivity	100	48.7 (37.0 - 60.5)
Specificity	100	73.0 (57.6 - 86.2)
PPV	100	74.5 (57.1 - 90.5)
NPV	100	48.6 (19.2 - 63.6)
**Test B**		
Sensitivity	83.3 (71.0 - 91.3)	98.1 (92.9 - 100)
Specificity	20.2 (12.7 - 30.3)	47.6 (40.4 - 96.5)
PPV	41.3 (32.6 - 50.6)	74.1 (61.2 - 99.3)
NPV	64.3 (44.1 - 80.7)	94.1 (77.2 - 100)
**Test C**		
Sensitivity	68.3 (54.9 - 79.4)	88.2 (63.4 - 100)
Specificity	55.1 (44.2 - 65.5)	97.7 (84.7 - 100)
PPV	50.6 (39.4 - 61.8)	98.4 (88.0 - 100)
NPV	72.1 (59.7 - 81.9)	84.3 (36.2 - 100)

* Gold standard model assumed that test A is perfect (100% sensitivity and 100% specificity; all patients with gold standard test positive are diseased and all patients with gold standard test negative are non-diseased). Values shown are estimated means with 95% confidence interval.

** Bayesian latent class model assumed that all tests evaluated are imperfect. Values shown are estimated median with 95% credible interval.

THINGS TO BE AWARE OF!!!

1) Results estimated by Bayesian LCM are reliable only when the chains in Bayesian LCM converged properly. Therefore, please check for the convergence before considering the result in the summary table.

2) Results estimated by Bayesian LCM are reliable only when the frequencies predicted by Bayesian LCM do fit with the observed data. Therefore, please check for the fitness of the model before considering the result in the summary table.

3) Results estimated by Bayesian LCM here should be used as a preliminary statistical analysis ONLY. For further usage, please consult experienced Bayesian statisticians for thorough analysis and confirmation.

2. Checking for convergence of Bayesian LCM

Please carefully evaluate histogram and tracing plots of prevalence, sensitivities, specificities, PPVs and NPVs to check for convergence of two chains generated by Bayesian LCM. **(Hide)**

WARNING!!!
Please ensure that chains do **CONVERGE!!!** The follow two examples illustrate what kind of convergency is acceptable and what is not acceptable.

Example 1

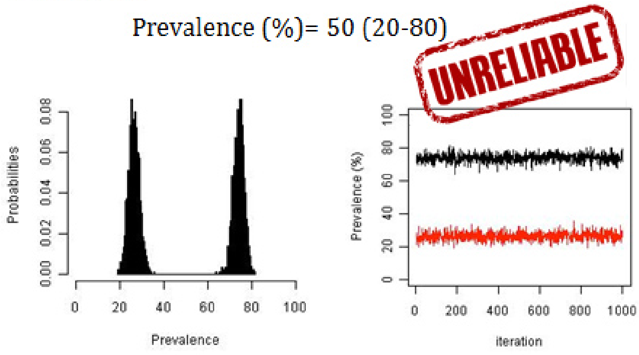

Example 2

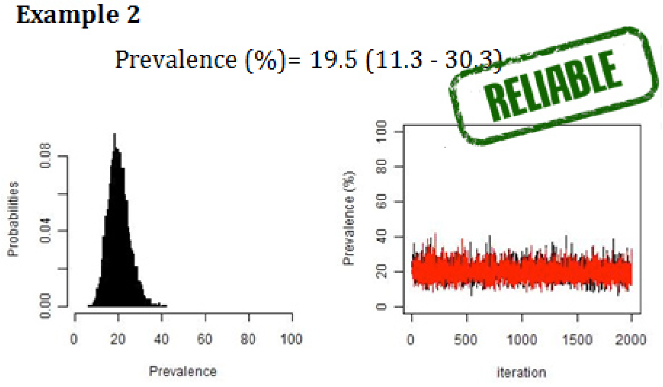

The **black line** represents chain 1 and **red line** represents chain 2. If the two chains do not converge (as in Example 1), the estimated parameters by the Bayesian model are **UNRELIABLE**.

There are many reasons for the chains not converged, please consult WinBUGS manual, standard textbooks of Bayesian statistics or experienced statisticians.

Prevalence (%)* = 60.6 (47.4 - 83.4)

Histogram	Tracing plots

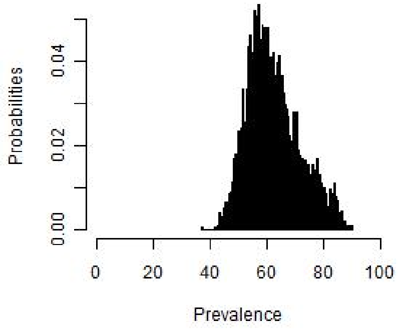

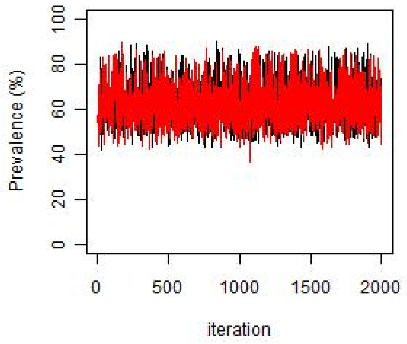

Sensitivity of Test A (%)* = 48.7 (37.0 - 60.5)

Histogram	Tracing plots

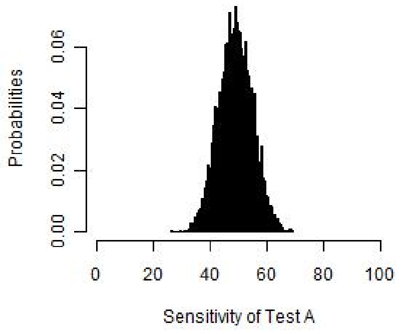

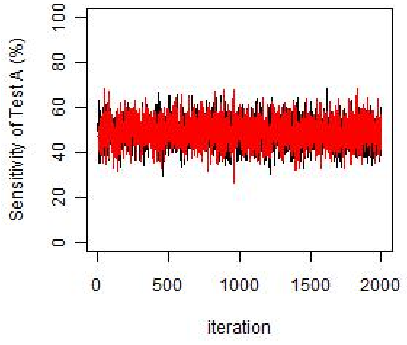

Specificity of Test A (%)* = 73.0 (57.6 - 86.2)

Histogram	Tracing plots

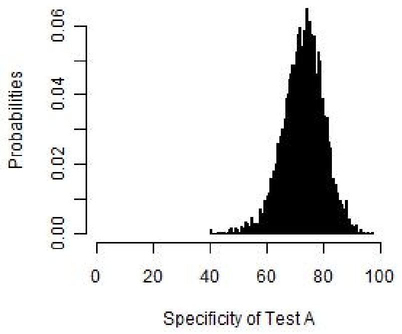

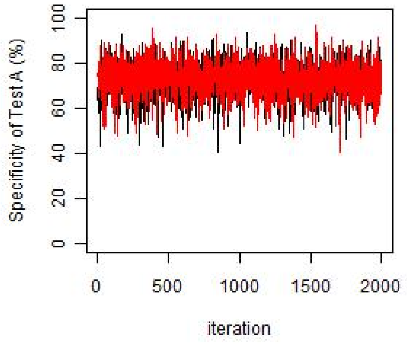

Sensitivity of Test B (%)* = 98.1 (92.9 - 100)

Histogram	Tracing plots

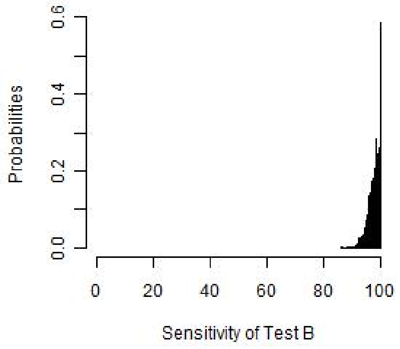	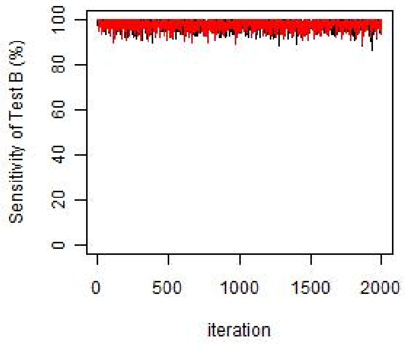

Specificity of Test B (%)* = 47.6 (40.4 - 96.5)	
Histogram	Tracing plots
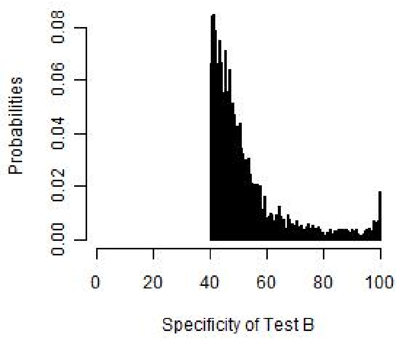	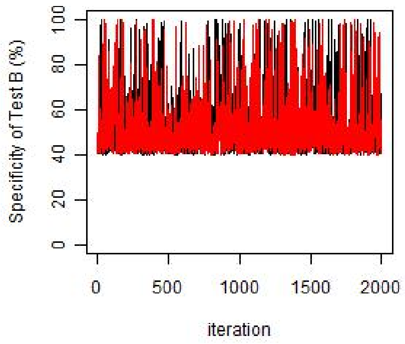

Sensitivity of Test C (%)* = 88.2 (63.4 - 100)	
Histogram	Tracing plots
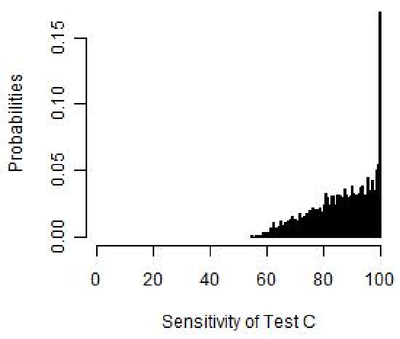	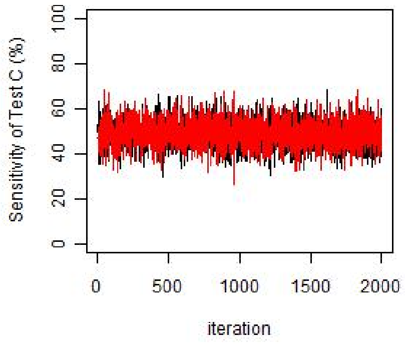

Specificity of Test C (%)* = 88.2 (63.4 - 100)	
Histogram	Tracing plots

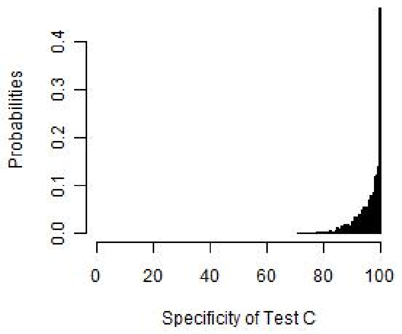

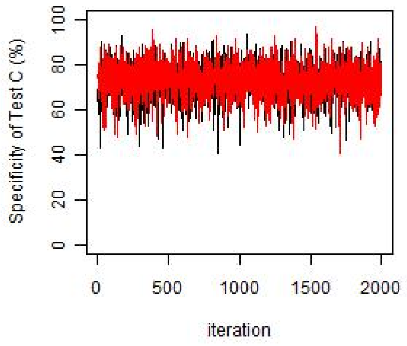

* Bayesian latent class model %

3. Checking for fitness of Bayesian LCM

Please carefully assess the agreement between "frequency observed" and "frequency predicted" using Bayesian p value and posterior predictive distribution of each profile. **(Hide)**

Profiles	Test A	Test B	Test C	Frequency observed	Frequency predicted	Bayesian p value *
111	Positive	Positive	Positive	39	37	0.444
110	Positive	Positive	Negative	11	13	0.718
101	Positive	Negative	Positive	2	1	0.330
011	Negative	Positive	Positive	40	40	0.534
100	Positive	Negative	Negative	8	7	0.467
010	Negative	Positive	Negative	31	27	0.271
001	Negative	Negative	Positive	0	1	1.000
000	Negative	Negative	Negative	18	20	0.654

* Bayesian p-value is the probability that replicate data (predicted frequency) from the Bayesian model were more extreme than the observed data. A Bayesian p-value close to 0 or 1 indicates that the observed result would be unlikely to be seen in replication of the data if the mode was true. This means that when Bayesian p-value is **close to 0.5 or exactly 0.5**, the Bayesian model describes the observed data very well.

Histogram

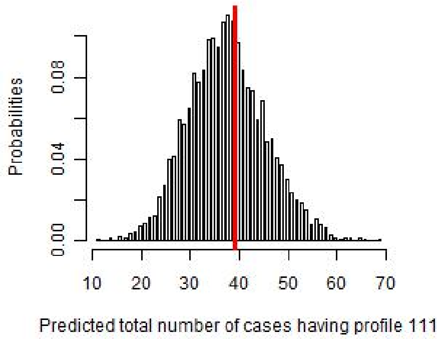

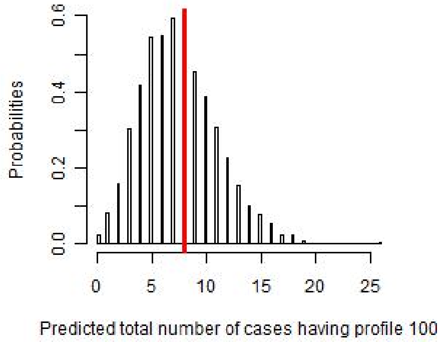

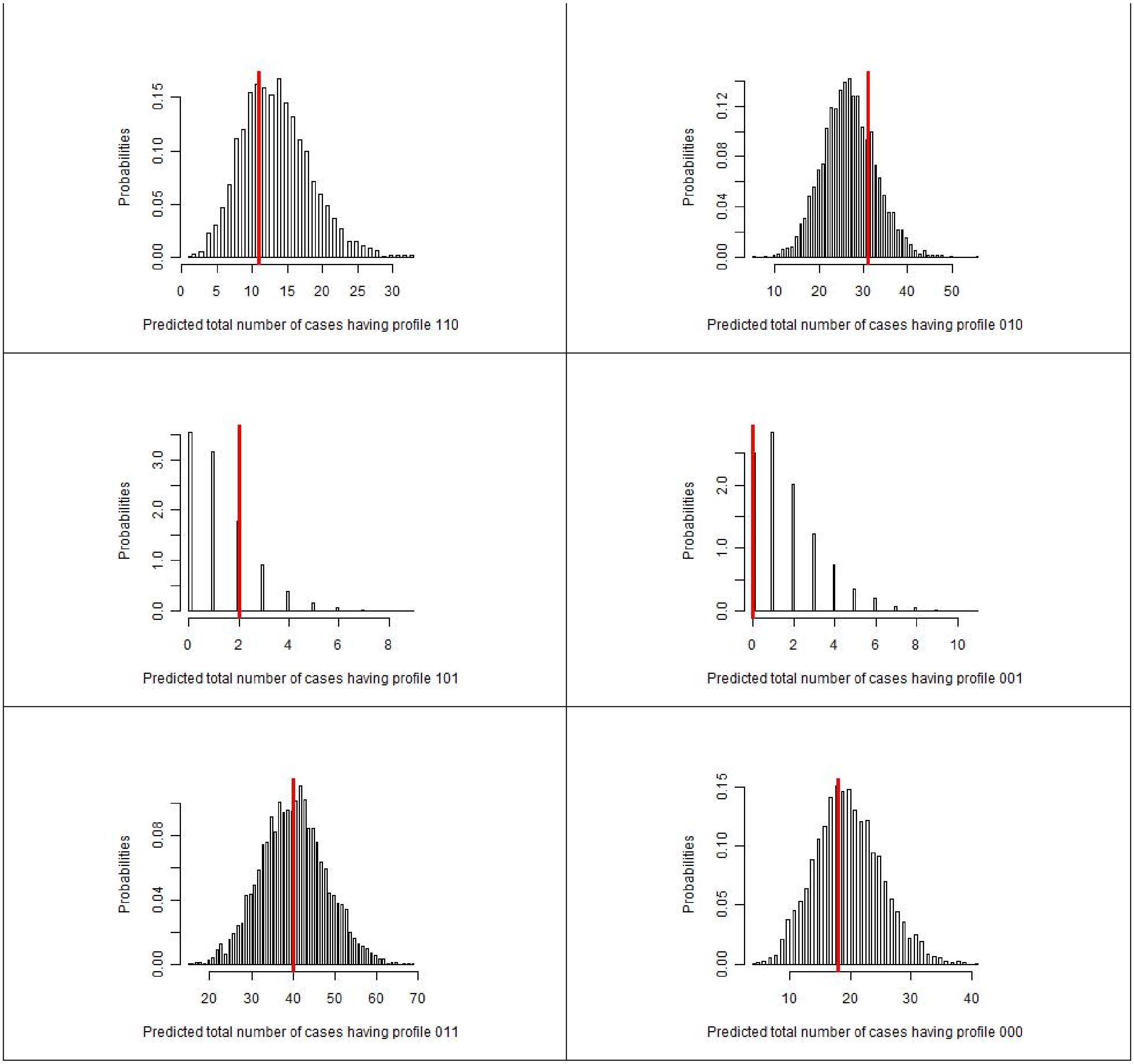

Red line represents the observed frequency of each test result profile, while the histograms illustrate the predictive posterior distribution of predicted frequency.

In each of the figures, dataset was replicated for **20000** times and selected only **2000** time (thin sampling equals to **10**) to assess the probability of observed frequencies, assuming the model was true.
