# Supplementary material for: Reevaluating the true diagnostic accuracy of dipstick tests to diagnose urinary tract infection using Bayesian latent class analysis
Source: PLoS One. 2020 Dec 31;15(12):e0244870. doi: 10.1371/journal.pone.0244870 (PMC7774958; doi:10.1371/journal.pone.0244870)
Supplement: S3 File — (PDF) [file pone.0244870.s003.pdf]

Result Page

Model Code : MODEL101

Model Name : The 2-tests in 2-populations Model (Simplified Interface)

Job ID : 20191117180942390

1. Summary Table

Prevalence, sensitivities, specificities, and positive and negative predictive values (PPV and NPV) estimated by using the conventional method (assuming that a test is perfect) and imperfect gold standard model (Bayesian latent class model). **(Hide)**

Parameters	Test A was assumed as a perfect gold standard (%)*	Bayesian latent class model (%) **
**Prevalence**		
Population 1	87.5 (76.3 - 94.1)	80.2 (62.5 - 94.2)
Population 2	76.5 (65.8 - 84.7)	49.8 (34.2 - 75.2)
**Test A**		
Sensitivity	100 (100 - 100)	97.8 (92.5 - 100)
Specificity	100 (100 - 100)	49.0 (40.5 - 90.0)
PPV	100 (100 - 100)	76.5 (62.3 - 97.8)
NPV	100 (100 - 100)	93.1 (76.0 - 99.9)
**Test B**		
Sensitivity	65.3 (56.0 - 73.6)	84.9 (65.5 - 99.8)
Specificity	92.9 (75.0 - 98.8)	98.2 (85.3 - 100)
PPV	97.5 (90.5 - 99.6)	98.8 (88.9 - 100)
NPV	38.2 (27.0 - 50.9)	79.0 (40.5 - 99.8)

* Conventional method assumed that test A is perfect (100% sensitivity and 100% specificity; all patients with gold standard test positive are diseased and all patients with gold standard test negative are non-diseased). Values shown are estimated means with 95% confidence interval.

** Bayesian latent class model does not assume that any test is perfect. Values shown are estimated median with 95% credible interval.

THINGS TO BE AWARE OF!!!

1) Results estimated by Bayesian LCM are reliable only when the chains in Bayesian LCM converged properly. Therefore, please check for the convergence before considering the result in the summary table.

2) Results estimated by Bayesian LCM are reliable only when the frequencies predicted by Bayesian LCM fit with the observed data. Therefore, please check for the fitness of the model before considering the result in the summary table.

2. Checking for convergence of Bayesian LCM

Please carefully evaluate histogram and tracing plots of prevalence, sensitivities, specificities, and PPVs and NPVs to check for convergence of two chains generated by Bayesian LCM. **(Hide)**

WARNING!!!

Please ensure that chains do **CONVERGE!!!** The follow two examples illustrate what kind of convergency is acceptable and what is not acceptable.

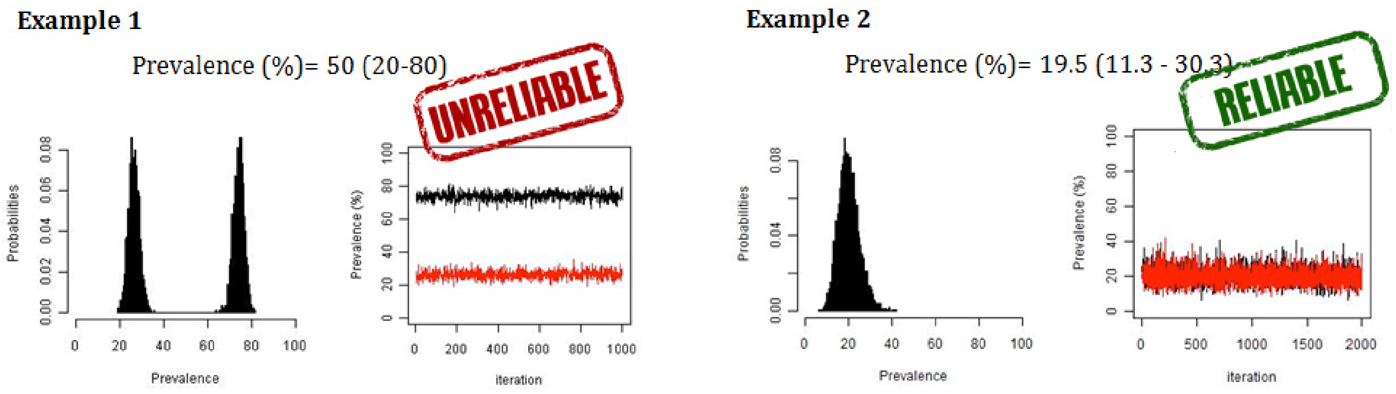

The **black line** represents chain 1 and **red line** represents chain 2. If the two chains do not converge (Example 1), the estimated parameters by the Bayesian model are **UNRELIABLE**.

There are many reasons for the chains not converged, please consult WinBUGS manual, standard textbooks of Bayesian statistics or experienced statisticians.

Prevalence of population 1 (%)* = 80.2 (62.5 - 94.2)	
Histogram	Tracing plots
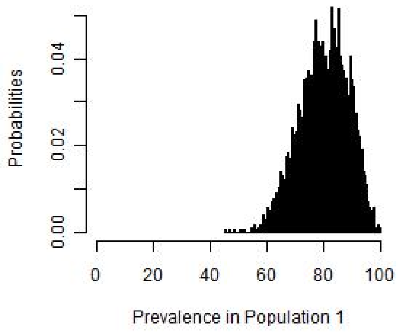	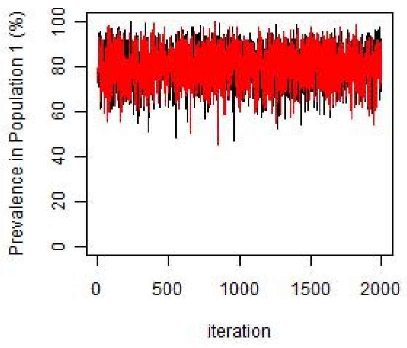
**Prevalence of population 2 (%)* = 49.8 (34.2 - 75.2)**	
Histogram	Tracing plots
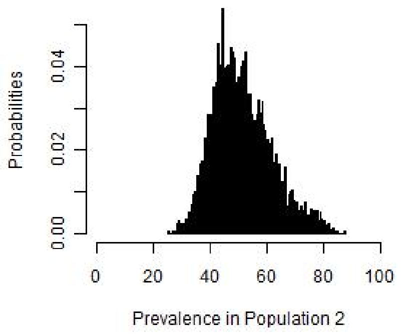	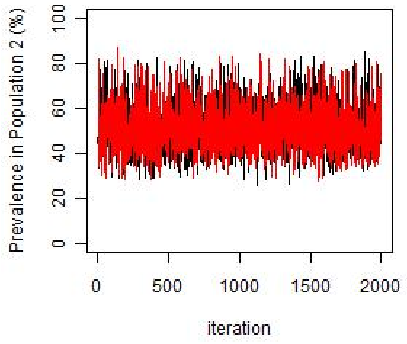

Sensitivity of Test A (%)* = 97.8 (92.5 - 100)	
Histogram	Tracing plots
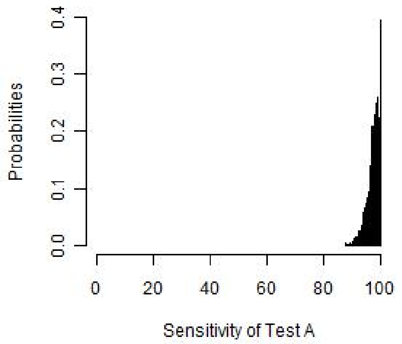	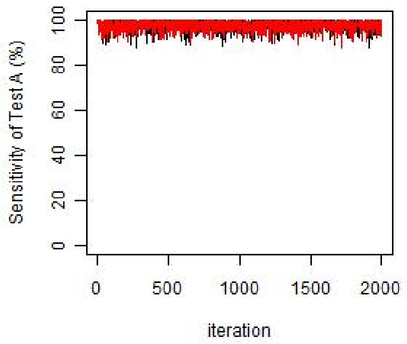
**Specificity of Test A (%)* = 49.0 (40.5 - 90.0)**	
Histogram	Tracing plots

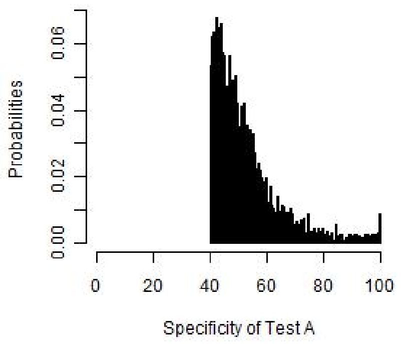

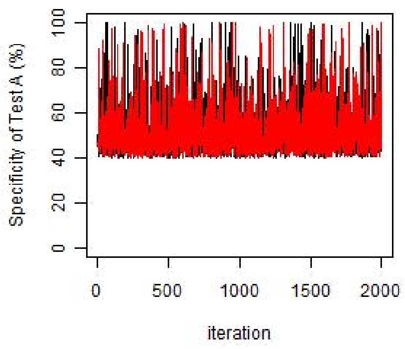

Sensitivity of Test B (%)* = 84.9 (65.5 - 99.8)

Histogram	Tracing plots

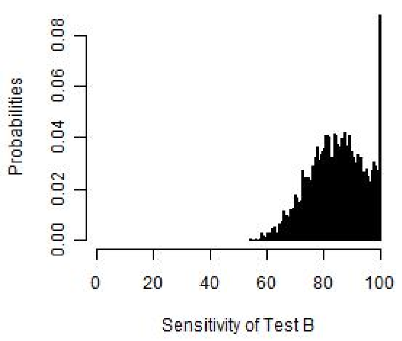

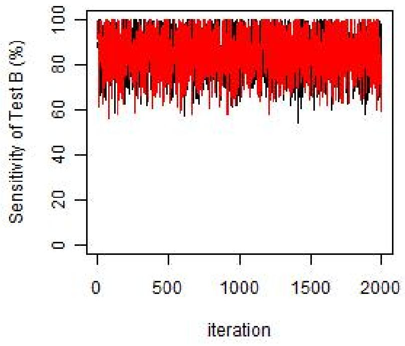

Specificity of Test B (%)* = 98.2 (85.3 - 100)

Histogram	Tracing plots

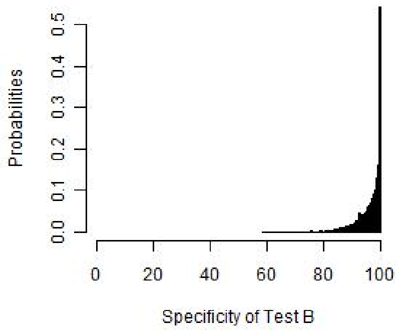

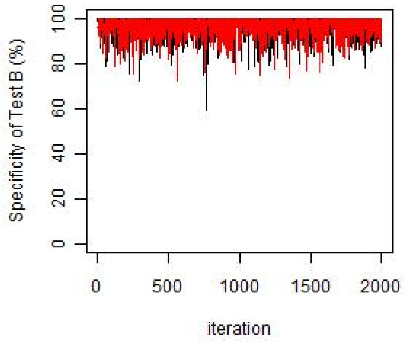

* Bayesian latent class model %

3. Checking for fitness of Bayesian LCM

Please carefully assess the agreement between "frequency observed" and "frequency predicted" using Bayesian p value and posterior predictive distribution of each profile. **(Hide)**

	Test A	Test B	Frequency observed	Frequency predicted	Bayesian p value *
Population 1	Positive	Positive	43	42	0.469
	Positive	Negative	13	14	0.629
	Negative	Positive	2	1	0.369
	Negative	Negative	6	6	0.581
Population 2	Positive	Positive	36	36	0.516
	Positive	Negative	29	27	0.383

	Negative	Positive	0	1	1.000
	Negative	Negative	20	20	0.567

* Bayesian p-value is the probability that replicate data (predicted frequency) from the Bayesian model were more extreme than the observed data. A Bayesian p-value close to 0 or 1 indicates that the observed result would be unlikely to be seen in replication of the data if the mode was true. This means that when Bayesian p-value is **close to 0.5 or exactly 0.5**, the Bayesian model describes the observed data very well. Values of frequency predicted should be close to values of frequency observed.

Histogram

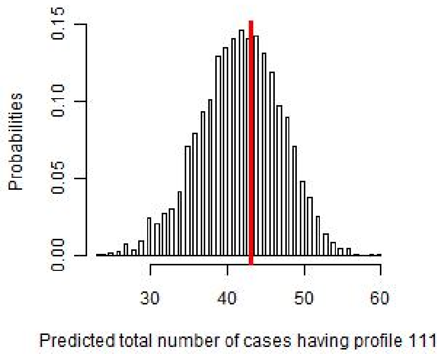

Population 1, Test A positive and Test B positive

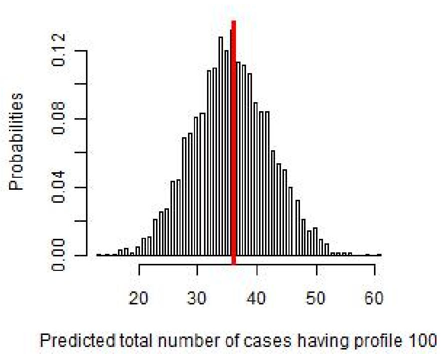

Population 2, Test A positive and Test B positive

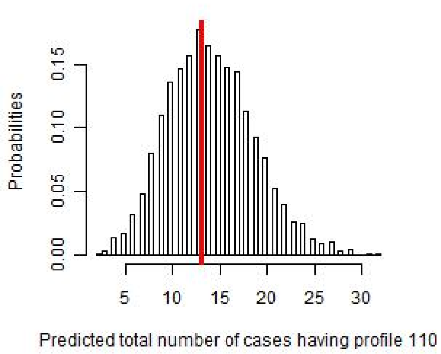

Population 1, Test A positive and Test B negative

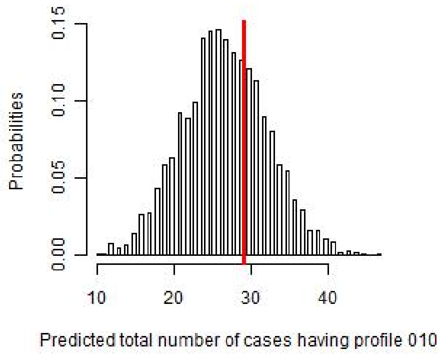

Population 2, Test A positive and Test B negative

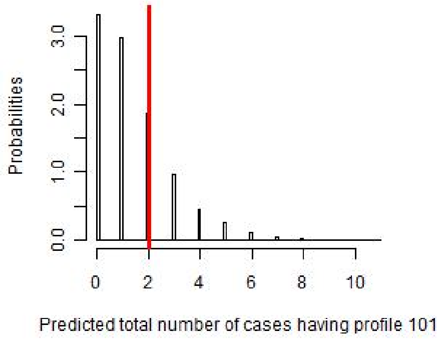

Population 1, Test A negative and Test B positive

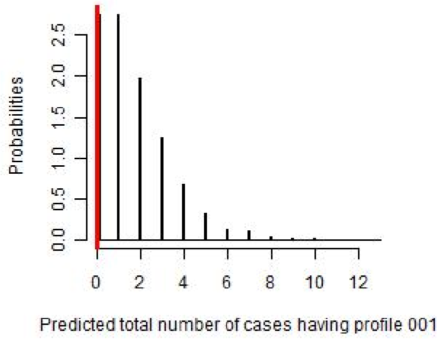

Population 2, Test A negative and Test B positive

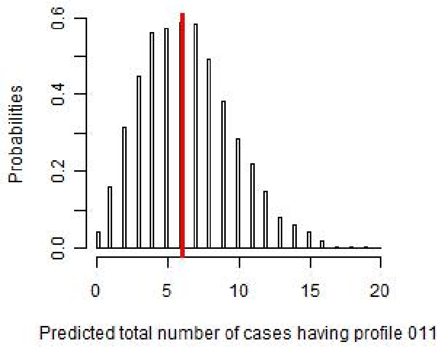

Population 1, Test A negative and Test B negative

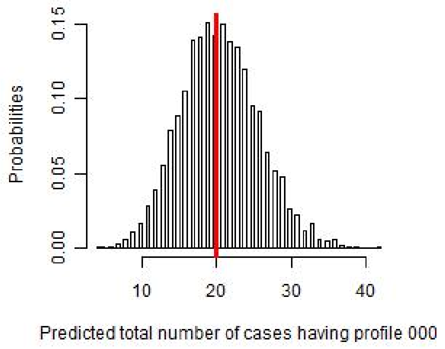

Population 2, Test A negative and Test B negative

Red line represents the observed frequency of each test result profile, while the histograms illustrate the predictive posterior distribution of predicted frequency.

In each of the figures, dataset was replicated for **20000** times and selected only **2000** time (thin sampling equals to **10**) to assess the probability of observed frequencies, assuming the model was true.
