# Supplementary material for: Reevaluating the true diagnostic accuracy of dipstick tests to diagnose urinary tract infection using Bayesian latent class analysis
Source: PLoS One. 2020 Dec 31;15(12):e0244870. doi: 10.1371/journal.pone.0244870 (PMC7774958; doi:10.1371/journal.pone.0244870)
Supplement: Attachment — Submitted filename: Response to reviewers.rtf [file pone.0244870.s005.rtf]

To
The Editorial Team, PLOS ONE
Dear Editors,
Thank you for giving us an opportunity to clarify regarding our submission PONE-D-20-16512. We would like to highlight that this manuscript is not a mere re-submission of our previously rejected manuscript PONE-D-18-33448R1. As we had made clear in our cover letter accompanying the present submission “We have considerably expanded our data analysis by incorporating Bayesian latent class models and substantially revised our manuscript”. We have done this to address the peer reviewers' comments on our previous submission — “the core concept of the study does not significantly add to the body of knowledge existent concerning this topic”; “as the manuscript exists, I do not feel this work is novel enough, nor has the impact of importance, for our clinical practice and care of patients.” 
In the present manuscript, we have employed Bayesian latent class analysis. To our knowledge, Bayesian latent class analysis has not been applied previously in the context of urinary tract infections. Kindly note that Bayesian latent class analysis is completely different from Bayesian approach to estimating post-test probability, which we had used in our earlier submission. Notably, we found that Bayesian latent class models indicate a clinically important improvement in the true diagnostic accuracy of urine dipstick testing for LE and nitrite. We sincerely believe that our present analysis is novel and has considerable implications for clinical practice and future research. In addition, we have also addressed other comments by the peer reviewers.   
Please find below a point by point response to the reviewers' comments on our previous submission (PONE-D-18-33448R1).
Best wishes & regards,
Deepanjali
Response to reviewers' comments
Reviewer #4: The manuscript presented is very well written, and well- constructed. Analyses are performed with rigor. Excellent points are made by the authors in all points. However, I am in agreement with previous reviewers; the core concept of the study does not significantly add to the body of knowledge existent concerning this topic.
The study takes a more in depth approach to analyses of dipstick UA (employing Bayesian analysis for example) compared to previous studies, and reviews in great detail the prior completed studies on the topic. It serves as an excellent literature summary for the topic as well for readers. However, the conclusions of the study do not significant impact the clinical care of patients in the ED, and there is not enough emphasis concerning the realistic implications of the conclusions.
The authors state: When a broader definition is assumed, the specificity and PPV are higher but not good enough to rule in the diagnosis. In settings where majority of patients present with high pre-test probability of UTI, dipstick tests cannot rule out the disease.
Do the authors advocate therefore that the UA dipstick is not essentially useful in any given clinical scenario? This is not the case. A rework of the clinical applicability of the UA dipstick may help the authors in convincing reviewers of the true merit of their work, but as the manuscript exists, I do not feel this work is novel enough, nor has the impact of importance, for our clinical practice and care of patients.
Response: We thank the reviewer for the encouraging comments. We agree with the reviewer that the conclusions that we drew in the study gives the impression as if the urine dipstick is not essentially useful in any given clinical scenario. As suggested by the reviewer we have done “a rework of the clinical applicability of the UA dipstick”. By application of Bayesian latent class models, we could come to the clinically meaningful conclusion that the true performance of urine dipsticks is substantially better than what is generally evident through conventional calculation of just sensitivity, specificity and predictive values. Thus, as stated before, we believe that our present work has novelty as well as direct clinical applicability. We again thank the reviewer for the constructive and critical comments.

Reviewer #5: This study is a prospective observational study aiming to evaluate diagnostic performance of urine dip stick in emergency department. Additionally, the authors tried to identify actual diagnostic performance of urine dip stick using pragmatic analysis. Regardless of major revision, this study is not accepted to publish following reasons.
Response: We have substantially modified the manuscript in this regard. For better understanding, we have retained the conventional/classical analysis and compare it with Bayesian latent class models, while the pragmatic analysis has been removed. 
[Major concerns]
1. Heterogeneous population. In this study, the authors included 57% DM and 62% AKI patients suggesting immunocompromised state due to DM or multi-organ failure by septic condition. Since DM mostly can cause immunocompromised state, DM-AKI patients frequently present clinical state such as no febrile condition or few pyuria and are also vulnerable to sepsis. These large difference of UTI severity and clinical manifestation masking possibly affect irrelevant conclusion regardless of the use of pragmatic analysis. Other important laboratory (WBC, PLT, procalcitonin etc.) and clinical (vital sign, mentality, GCS score etc.) data should be provided to evaluate the balance of severity in both gender groups.
2. Unclear reports for the use of antibiotics or antipyretic drugs. Absence of pyuria easily can occur when using antibiotics. The use of antipyrectic drugs also can induce no fever. Therefore, the authors should state the use of these drugs and regarding medical conditions. Furthermore, the authors did not state any timing of administration of antibiotics for UTI in urine sampling. As you know, urine sampling after administration of antibiotics also cause absence of pyuria.
Response to #1&2: We agree with the reviewer that our study population included a good proportion of patients with either clinically severe UTI or with co-morbidities. This is reflective of the patient population in whom these tests would be used in real-life settings. In the Discussion, we do explain the 'spectrum effect' that may influence the performance of dipsticks in different patient populations. As rightly pointed out by the reviewer, microscopic pyuria could be falsely negative in cases where antibiotic therapy is initiated before sending urine microscopy. The same is true for urine culture too. Thus, microscopic pyuria or urine culture are imperfect gold standards for comparison of dipstick performance. This is precisely the reason why latent class analysis could help, and hence we have made use of it in our re-analysis.
We would also like to point out that we had included data on the WBC count in Table 1. However, we do not feel it is necessary to include data on vital signs and GCS scores since it has no direct implications for the content matter.      

3. Unclear definitions. The authors are needed to clearly define variables using objective standard for fever or AKI. It is very important to report what standard for body temperature to define fever, what kind of body temperature measurement device was used, and the standard of GFR to define AKI.
Response: We have defined AKI in the revised manuscript (Line 196). Digital thermometers have been used to measure temperature. We can include this information in the manuscript if deemed necessary by the Editor.   

4. Small effect size. This study was performed in single center for 9 months in Southern India. Therefore, the failure of obtaining wide representativeness is necessarily significant limitation of this study. The authors also should tone down in conclusion.
Response: It is not very clear to us what the reviewer meant by the term 'effect size'. We surmise that he/she is referring to the generalizability and external validity of our findings. We do agree that our study was conducted in a single centre only in the emergency setting. In view of this limitation, we do write in Discussion (Lines 298-301) that “The present study demonstrates the applicability and validity of using Bayesian LCMs in the context of UTI diagnostics. We suggest that the true diagnostic accuracy and utility of urine dipstick tests in various practice settings should be re-evaluated using Bayesian LCMs.”
