# Supplementary material for: Reevaluating the true diagnostic accuracy of dipstick tests to diagnose urinary tract infection using Bayesian latent class analysis
Source: PLoS One. 2020 Dec 31;15(12):e0244870. doi: 10.1371/journal.pone.0244870 (PMC7774958; doi:10.1371/journal.pone.0244870)
Supplement: Attachment — Submitted filename: Response to reviewers.docx [file pone.0244870.s006.docx]

**Point-by-point response to academic editors’ comments**

**Comment:** I found the presented study very interesting. However, with its current form, I am afraid that it won't receive favorable review from Bayesian experts.

**Response:** We thank Dr Dong for finding our study interesting, and we hope to address the concerns raised by him in our revised manuscript.

**Comment:** In particular, I found the Method section "Bayesian latent class analysis" did not sufficiently describe how exactly the Bayesian method was constructed. In particular, I found the Method section "Bayesian latent class analysis" did not sufficiently describe how exactly the Bayesian method was constructed. For example, what's exactly the likelihood function? what's the posterior model? The only thing mentioned is prior for all parameters "We used the simplified interface which assumes a non-informative prior distribution for all parameters (beta distribution (0.5,0.5)), except that specificity was set between 0.4 and 1.0 to prevent estimating test accuracy the other way around [18]." But without clearly describing the model, we won't even know what "parameters" you are referring to.

**Response:** We thank Dr Dong for the helpful comments to improve our manuscript. We have substantially modified our Methods section now. Upon reviewing this section in our previous submission, we realized that we made a typological error in citing the major reference for the methods section, “*Lim C, Wannapinij P, White L, Day NPJ, Cooper BS, et al. (2013) Using a Web-Based Application to Define the Accuracy of Diagnostic Tests When the Gold Standard Is Imperfect. PLoS ONE 8(11): e79489. doi:10.1371/journal.pone.0079489*”, which should have been reference [18]. We apologize for this error. We have now included details on how the models were constructed, likelihood function, parameters, and assumptions in the revised Methods section (Lines 152 to 171 of revised manuscript). We have included more details on likelihood function and posterior model in the Supplementary File (S1 File).

We sincerely hope that you find these modifications satisfactory. We will be happy to make further modifications if deemed necessary.

Best regards,

Deepanjali
